# (*E*)-3-(1,3-Benzodioxol-5-yl)-1-{4-[bis­(4-meth­oxy­phen­yl)meth­yl]piperazin-1-yl}prop-2-en-1-one

**DOI:** 10.1107/S1600536812004345

**Published:** 2012-02-10

**Authors:** Yan Zhong, Bin Wu

**Affiliations:** a School of Chemistry and Chemical Engineering, Southeast University, Sipailou No. 2 Nanjing, Nanjing 210096, People’s Republic of China; b School of Pharmacy, Nanjing Medical University, Hanzhong Road No. 140 Nanjing, Nanjing 210029, People’s Republic of China

## Abstract

There are two crystallographically independent mol­ecules in the asymmetric unit of the title compound, C_29_H_30_N_2_O_5_, each having an *E* conformation about the C=C double bond. The dihedral angles between the meth­oxy­benzene rings in the two mol­ecules are 71.39 (17) and 68.64 (17)°. In the crystal, mol­ecules are linked by C—H⋯O inter­actions.

## Related literature

For related structures and background to cinnamic acid derivatives, see: Teng *et al.* (2011 ▶); Zhong *et al.* (2012 ▶). For further synthetic details, see: Wu *et al.* (2008 ▶).
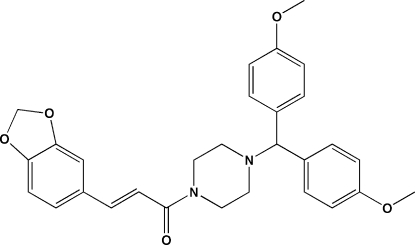


## Experimental

### 

#### Crystal data

C_29_H_30_N_2_O_5_
*M* *_r_* = 486.50
Triclinic, 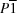

*a* = 12.188 (2) Å
*b* = 12.589 (3) Å
*c* = 17.102 (3) Å
α = 76.52 (3)°
β = 84.10 (3)°
γ = 89.95 (3)°
*V* = 2537.5 (9) Å^3^
*Z* = 4
Mo *K*α radiation
μ = 0.09 mm^−1^
*T* = 293 K
0.30 × 0.20 × 0.20 mm

#### Data collection

Enraf–Nonius CAD-4 diffractometer
Absorption correction: ψ scan (North *et al.*, 1968 ▶) *T*_min_ = 0.974, *T*_max_ = 0.983
9818 measured reflections
9340 independent reflections
5434 reflections with *I* > 2σ(*I*)
*R*_int_ = 0.015
3 standard reflections every 200 reflections intensity decay: 1%

#### Refinement

*R*[*F* ^2^ > 2σ(*F* ^2^)] = 0.068
*wR*(*F* ^2^) = 0.193
*S* = 1.00
9340 reflections
649 parameters
H-atom parameters constrained
Δρ_max_ = 0.21 e Å^−3^
Δρ_min_ = −0.21 e Å^−3^

### 

Data collection: *CAD-4 EXPRESS* (Enraf–Nonius, 1994 ▶); cell refinement: *CAD-4 EXPRESS*; data reduction: *XCAD4* (Harms & Wocadlo, 1995 ▶); program(s) used to solve structure: *SHELXS97* (Sheldrick, 2008 ▶); program(s) used to refine structure: *SHELXL97* (Sheldrick, 2008 ▶); molecular graphics: *SHELXL97*; software used to prepare material for publication: *SHELXL97*.

## Supplementary Material

Crystal structure: contains datablock(s) I, global. DOI: 10.1107/S1600536812004345/hb6623sup1.cif

Structure factors: contains datablock(s) I. DOI: 10.1107/S1600536812004345/hb6623Isup2.hkl

Supplementary material file. DOI: 10.1107/S1600536812004345/hb6623Isup3.cml

Additional supplementary materials:  crystallographic information; 3D view; checkCIF report

## Figures and Tables

**Table 1:** Hydrogen-bond geometry (Å, °)

*D*—H⋯*A*	*D*—H	H⋯*A*	*D*⋯*A*	*D*—H⋯*A*
C29—H29*A*⋯O10^i^	0.97	2.52	3.134 (5)	121
C44—H44*A*⋯O6^ii^	0.96	2.59	3.322 (5)	134
C47—H47*B*⋯O4^ii^	0.97	2.48	3.353 (5)	149
C53—H53*A*⋯O5^iii^	0.93	2.35	3.266 (4)	169

Symmetry codes: (i) 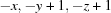; (ii) 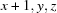; (iii) 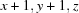.

**Fig. 1.**
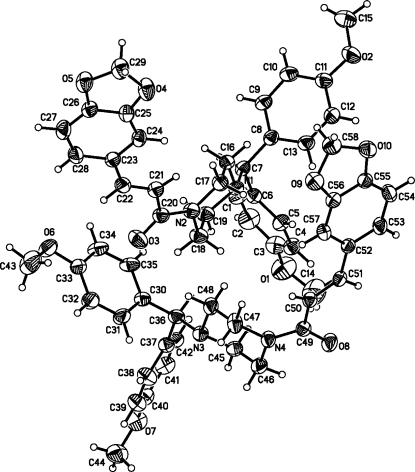
The molecular structure of (I) with displacement ellipsoids for non-H drawn at 70% probability level.

**Fig. 2.**
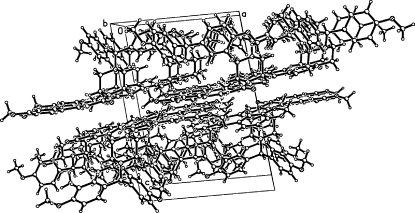
Packing diagram of the title compound.

## supplementary crystallographic information

### Comment

As a continuation of our study of cinnamic acid derivatives (Teng *et al.*, 2011; Zhong *et al.*, 2012), we present here the title compound (I). In (I) (Fig. 1), all bond lengths and angles are normal and correspond to those observed in related compounds (Teng *et al.*, 2011; Zhong *et al.*, 2012). The asymmetric unit of the title compound contains two crystallographically independent molecules with different absolute configurations. The dihedral angle between the methoxybenzene rings in the two molecules are 71.39 (17) and 68.64 (17)°.

### Experimental

The synthesis follows the method of Wu *et al.* (2008). The title compound was prepared by stirring a mixture of (*E*)(benzo[*d*][1,3]dioxol-5-yl) acrylic acid (0.769 g; 4 mmol), thionyl chloride (2 ml) and dichloromethane (30 ml) for 6 h at room temperature. The solvent was removed under reduced pressure. The residue was dissolved in acetone (15 ml) and reacted with 1-(bis(4-methoxyphenyl)methyl)iperazine (1.874 g; 6 mmol) in the presence of triethylamine (5 ml) for 12 h at room temperature. The resultant mixture was cooled. The solid, (*E*)-3-(benzo[*d*][1,3]dioxol-5-yl)-1-(4-(bis(4- methoxyphenyl)methyl)piperazin-1-yl)prop-2-en-1-one obtained was filtered and was recrystallized from ethanol. The pale-yellow blocks were grown from ethanol:ethyl acetate (1:1) solution by a slow evaporation at room temperature.

### Refinement

All non-hydrogen atoms were refined anisotropically. All hydrogen atoms were positioned geometrically with C—H distances ranging from 0.93 Å to 0.98 Å and refined as riding on their parent atoms with *U*ĩso~(H) = 1.2 or 1.5*U*~eq~ of the carrier atom.

### Crystal data

**Table:**

C_29_H_30_N_2_O_5_	*Z* = 4
*M**_r_* = 486.50	*F*(000) = 1032
Triclinic, *P*1	*D*_x_ = 1.274 Mg m^−^^3^
Hall symbol: -P 1	Mo *K*α radiation, λ = 0.71073 Å
*a* = 12.188 (2) Å	Cell parameters from 25 reflections
*b* = 12.589 (3) Å	θ = 9–13°
*c* = 17.102 (3) Å	µ = 0.09 mm^−^^1^
α = 76.52 (3)°	*T* = 293 K
β = 84.10 (3)°	Block, pale-yellow
γ = 89.95 (3)°	0.30 × 0.20 × 0.20 mm
*V* = 2537.5 (9) Å^3^	

### Data collection

**Table:**

Enraf–Nonius CAD-4 diffractometer	5434 reflections with *I* > 2σ(*I*)
Radiation source: fine-focus sealed tube	*R*_int_ = 0.015
Graphite monochromator	θ_max_ = 25.4°, θ_min_ = 1.2°
ω/2θ scans	*h* = −14→0
Absorption correction: ψ scan (North *et al.*, 1968)	*k* = −15→15
*T*_min_ = 0.974, *T*_max_ = 0.983	*l* = −20→20
9818 measured reflections	3 standard reflections every 200 reflections
9340 independent reflections	intensity decay: 1%

### Refinement

**Table:**

Refinement on *F*^2^	Primary atom site location: structure-invariant direct methods
Least-squares matrix: full	Secondary atom site location: difference Fourier map
*R*[*F*^2^ > 2σ(*F*^2^)] = 0.068	Hydrogen site location: inferred from neighbouring sites
*wR*(*F*^2^) = 0.193	H-atom parameters constrained
*S* = 1.00	*w* = 1/[σ^2^(*F*_o_^2^) + (0.1*P*)^2^ + 0.5*P*] where *P* = (*F*_o_^2^ + 2*F*_c_^2^)/3
9340 reflections	(Δ/σ)_max_ < 0.001
649 parameters	Δρ_max_ = 0.21 e Å^−^^3^
0 restraints	Δρ_min_ = −0.21 e Å^−^^3^

### Special details

**Table:**

Geometry. All e.s.d.'s (except the e.s.d. in the dihedral angle between two l.s. planes) are estimated using the full covariance matrix. The cell e.s.d.'s are taken into account individually in the estimation of e.s.d.'s in distances, angles and torsion angles; correlations between e.s.d.'s in cell parameters are only used when they are defined by crystal symmetry. An approximate (isotropic) treatment of cell e.s.d.'s is used for estimating e.s.d.'s involving l.s. planes.
Refinement. Refinement of *F*^2^ against ALL reflections. The weighted *R*-factor *wR* and goodness of fit *S* are based on *F*^2^, conventional *R*-factors *R* are based on *F*, with *F* set to zero for negative *F*^2^. The threshold expression of *F*^2^ > σ(*F*^2^) is used only for calculating *R*-factors(gt) *etc*. and is not relevant to the choice of reflections for refinement. *R*-factors based on *F*^2^ are statistically about twice as large as those based on *F*, and *R*- factors based on ALL data will be even larger.

### Fractional atomic coordinates and isotropic or equivalent isotropic displacement parameters (Å^2^)

**Table:**

	*x*	*y*	*z*	*U*_iso_*/*U*_eq_	
N1	0.55160 (19)	0.55413 (18)	0.24848 (14)	0.0487 (6)	
O1	1.0307 (2)	0.7745 (3)	0.1321 (2)	0.1103 (10)	
C1	0.7676 (3)	0.6823 (3)	0.0918 (2)	0.0724 (10)	
H1A	0.7386	0.6647	0.0481	0.087*	
N2	0.4762 (2)	0.3419 (2)	0.34069 (16)	0.0588 (7)	
O2	0.3170 (2)	1.0136 (2)	0.14123 (16)	0.0865 (8)	
C2	0.8757 (3)	0.7169 (3)	0.0836 (2)	0.0850 (11)	
H2A	0.9186	0.7222	0.0345	0.102*	
O3	0.50956 (19)	0.16387 (18)	0.35334 (16)	0.0796 (7)	
C3	0.9214 (3)	0.7436 (3)	0.1462 (2)	0.0697 (10)	
O4	−0.1208 (2)	0.15508 (19)	0.43410 (17)	0.0843 (8)	
C4	0.8567 (3)	0.7361 (3)	0.2173 (2)	0.0627 (9)	
H4A	0.8861	0.7552	0.2604	0.075*	
O5	−0.16342 (19)	−0.00103 (19)	0.39582 (16)	0.0783 (7)	
C5	0.7486 (3)	0.7009 (2)	0.22616 (18)	0.0563 (8)	
H5A	0.7066	0.6955	0.2755	0.068*	
C6	0.7004 (2)	0.6730 (2)	0.16372 (17)	0.0504 (7)	
C7	0.5795 (2)	0.6381 (2)	0.17202 (17)	0.0520 (7)	
H7A	0.5688	0.6042	0.1272	0.062*	
C8	0.5065 (2)	0.7357 (2)	0.16465 (17)	0.0502 (7)	
C9	0.4540 (3)	0.7735 (3)	0.09669 (19)	0.0656 (9)	
H9A	0.4611	0.7348	0.0564	0.079*	
C10	0.3916 (3)	0.8654 (3)	0.0851 (2)	0.0743 (10)	
H10A	0.3586	0.8887	0.0375	0.089*	
C11	0.3782 (3)	0.9229 (3)	0.1447 (2)	0.0608 (8)	
C12	0.4293 (3)	0.8858 (3)	0.2137 (2)	0.0628 (8)	
H12A	0.4218	0.9240	0.2544	0.075*	
C13	0.4903 (3)	0.7947 (3)	0.22343 (18)	0.0588 (8)	
H13A	0.5224	0.7709	0.2713	0.071*	
C16	0.4343 (2)	0.5216 (2)	0.26006 (19)	0.0533 (8)	
H16A	0.4163	0.4928	0.2149	0.064*	
H16B	0.3896	0.5851	0.2614	0.064*	
C14	1.0783 (4)	0.8160 (5)	0.1897 (4)	0.133 (2)	
H14A	1.1547	0.8340	0.1717	0.200*	
H14B	1.0725	0.7621	0.2401	0.200*	
H14C	1.0406	0.8805	0.1971	0.200*	
C17	0.4079 (3)	0.4360 (2)	0.3378 (2)	0.0581 (8)	
H17A	0.4189	0.4674	0.3832	0.070*	
H17B	0.3310	0.4133	0.3429	0.070*	
C15	0.2702 (4)	1.0603 (4)	0.0683 (3)	0.1136 (16)	
H15A	0.2292	1.1234	0.0747	0.170*	
H15B	0.2219	1.0075	0.0562	0.170*	
H15C	0.3281	1.0815	0.0248	0.170*	
C18	0.5928 (2)	0.3711 (3)	0.3251 (2)	0.0653 (9)	
H18A	0.6350	0.3067	0.3215	0.078*	
H18B	0.6158	0.3984	0.3695	0.078*	
C19	0.6159 (2)	0.4572 (3)	0.2474 (2)	0.0601 (8)	
H19A	0.6938	0.4773	0.2390	0.072*	
H19B	0.5987	0.4275	0.2025	0.072*	
C20	0.4431 (3)	0.2357 (3)	0.35612 (19)	0.0564 (8)	
C21	0.3225 (2)	0.2102 (2)	0.37468 (18)	0.0542 (8)	
H21A	0.2760	0.2612	0.3912	0.065*	
C22	0.2806 (3)	0.1167 (2)	0.36819 (19)	0.0588 (8)	
H22A	0.3309	0.0664	0.3550	0.071*	
C23	0.1643 (3)	0.0825 (2)	0.37921 (18)	0.0534 (8)	
C24	0.0798 (3)	0.1440 (2)	0.40747 (19)	0.0565 (8)	
H24A	0.0958	0.2076	0.4234	0.068*	
C25	−0.0261 (3)	0.1082 (2)	0.41086 (18)	0.0546 (8)	
C26	−0.0519 (3)	0.0146 (2)	0.38868 (19)	0.0565 (8)	
C27	0.0272 (3)	−0.0484 (3)	0.3627 (2)	0.0750 (10)	
H27A	0.0095	−0.1130	0.3488	0.090*	
C28	0.1363 (3)	−0.0123 (3)	0.3577 (2)	0.0683 (9)	
H28A	0.1923	−0.0535	0.3393	0.082*	
C29	−0.2091 (3)	0.0876 (3)	0.4238 (3)	0.0796 (11)	
H29A	−0.2543	0.0614	0.4748	0.096*	
H29B	−0.2552	0.1288	0.3849	0.096*	
O6	0.7706 (2)	0.0983 (2)	0.08851 (15)	0.0784 (7)	
O7	1.49740 (19)	0.3416 (2)	0.07875 (14)	0.0782 (7)	
O8	0.99144 (18)	0.66771 (19)	0.42440 (15)	0.0725 (7)	
O9	0.35558 (17)	0.62973 (18)	0.45580 (15)	0.0697 (6)	
O10	0.31739 (18)	0.81125 (19)	0.43670 (15)	0.0721 (6)	
N3	1.02818 (19)	0.40906 (19)	0.25906 (15)	0.0528 (6)	
N4	0.96061 (19)	0.5108 (2)	0.38878 (16)	0.0573 (7)	
C30	0.9733 (2)	0.3241 (2)	0.15224 (18)	0.0531 (7)	
C31	0.9568 (3)	0.2196 (3)	0.2009 (2)	0.0746 (11)	
H31A	0.9916	0.2006	0.2480	0.090*	
C32	0.8902 (3)	0.1430 (3)	0.1817 (2)	0.0746 (10)	
H32A	0.8801	0.0740	0.2162	0.089*	
C33	0.8390 (3)	0.1674 (3)	0.1126 (2)	0.0592 (8)	
C34	0.8552 (3)	0.2700 (3)	0.0629 (2)	0.0710 (10)	
H34A	0.8222	0.2877	0.0150	0.085*	
C35	0.9198 (3)	0.3467 (3)	0.0833 (2)	0.0651 (9)	
H35A	0.9276	0.4163	0.0495	0.078*	
C36	1.0476 (2)	0.4080 (2)	0.17213 (18)	0.0538 (8)	
H36A	1.0288	0.4800	0.1407	0.065*	
C37	1.1677 (2)	0.3903 (2)	0.14871 (17)	0.0514 (7)	
C38	1.2193 (3)	0.2976 (3)	0.1841 (2)	0.0664 (9)	
H38A	1.1789	0.2452	0.2239	0.080*	
C39	1.3285 (3)	0.2784 (3)	0.1633 (2)	0.0657 (9)	
H39A	1.3606	0.2147	0.1895	0.079*	
C40	1.3900 (3)	0.3537 (3)	0.10347 (18)	0.0571 (8)	
C41	1.3401 (3)	0.4478 (3)	0.06647 (19)	0.0643 (9)	
H41A	1.3803	0.4996	0.0259	0.077*	
C42	1.2323 (3)	0.4654 (3)	0.08904 (18)	0.0590 (8)	
H42A	1.2007	0.5299	0.0637	0.071*	
C43	0.7375 (5)	−0.0016 (3)	0.1443 (3)	0.127 (2)	
H43A	0.6903	−0.0427	0.1199	0.191*	
H43B	0.6982	0.0135	0.1919	0.191*	
H43C	0.8015	−0.0432	0.1586	0.191*	
C44	1.5496 (3)	0.2451 (4)	0.1164 (3)	0.0907 (12)	
H44A	1.6251	0.2470	0.0937	0.136*	
H44B	1.5121	0.1825	0.1078	0.136*	
H44C	1.5469	0.2406	0.1734	0.136*	
C45	1.0958 (2)	0.4951 (3)	0.2769 (2)	0.0576 (8)	
H45A	1.0779	0.5654	0.2439	0.069*	
H45B	1.1732	0.4824	0.2633	0.069*	
C46	1.0763 (3)	0.4974 (3)	0.3647 (2)	0.0647 (9)	
H46A	1.1010	0.4298	0.3974	0.078*	
H46B	1.1191	0.5571	0.3742	0.078*	
C47	0.8916 (3)	0.4280 (3)	0.3697 (2)	0.0673 (9)	
H47A	0.8145	0.4425	0.3827	0.081*	
H47B	0.9073	0.3567	0.4022	0.081*	
C48	0.9127 (2)	0.4279 (3)	0.2814 (2)	0.0630 (9)	
H48A	0.8673	0.3712	0.2700	0.076*	
H48B	0.8920	0.4976	0.2491	0.076*	
C49	0.9251 (2)	0.5981 (2)	0.41624 (18)	0.0523 (7)	
C50	0.8053 (2)	0.6090 (2)	0.43409 (18)	0.0507 (7)	
H50A	0.7599	0.5468	0.4464	0.061*	
C51	0.7610 (2)	0.7044 (2)	0.43307 (17)	0.0505 (7)	
H51A	0.8106	0.7622	0.4284	0.061*	
C52	0.6441 (2)	0.7322 (2)	0.43834 (17)	0.0463 (7)	
C53	0.6168 (3)	0.8402 (2)	0.4283 (2)	0.0611 (9)	
H53A	0.6734	0.8920	0.4218	0.073*	
C54	0.5091 (3)	0.8758 (3)	0.4273 (2)	0.0670 (9)	
H54A	0.4927	0.9493	0.4212	0.080*	
C55	0.4290 (2)	0.7981 (3)	0.43584 (18)	0.0520 (7)	
C56	0.4537 (2)	0.6898 (2)	0.44671 (17)	0.0485 (7)	
C57	0.5584 (2)	0.6537 (2)	0.44787 (17)	0.0510 (7)	
H57A	0.5733	0.5798	0.4547	0.061*	
C58	0.2750 (3)	0.7087 (3)	0.4303 (2)	0.0738 (10)	
H58C	0.2621	0.7113	0.3749	0.089*	
H58A	0.2059	0.6903	0.4644	0.089*	

### Atomic displacement parameters (Å^2^)

**Table:**

	*U*^11^	*U*^22^	*U*^33^	*U*^12^	*U*^13^	*U*^23^
N1	0.0479 (14)	0.0481 (13)	0.0491 (14)	−0.0036 (11)	−0.0107 (11)	−0.0072 (11)
O1	0.077 (2)	0.139 (3)	0.108 (2)	−0.0300 (18)	0.0070 (17)	−0.021 (2)
C1	0.073 (2)	0.099 (3)	0.047 (2)	−0.004 (2)	0.0015 (17)	−0.0225 (18)
N2	0.0505 (15)	0.0496 (15)	0.0735 (18)	−0.0089 (12)	−0.0131 (13)	−0.0059 (12)
O2	0.106 (2)	0.0724 (16)	0.0827 (18)	0.0310 (15)	−0.0291 (15)	−0.0137 (13)
C2	0.076 (3)	0.113 (3)	0.061 (2)	−0.005 (2)	0.015 (2)	−0.018 (2)
O3	0.0619 (15)	0.0553 (14)	0.115 (2)	0.0031 (12)	−0.0060 (14)	−0.0085 (13)
C3	0.050 (2)	0.074 (2)	0.074 (3)	−0.0135 (17)	0.0056 (18)	−0.0009 (18)
O4	0.0647 (16)	0.0678 (15)	0.124 (2)	−0.0184 (13)	0.0136 (14)	−0.0388 (15)
C4	0.064 (2)	0.0597 (19)	0.063 (2)	−0.0105 (16)	−0.0100 (17)	−0.0098 (16)
O5	0.0618 (15)	0.0709 (15)	0.106 (2)	−0.0196 (12)	0.0019 (13)	−0.0329 (14)
C5	0.058 (2)	0.0652 (19)	0.0434 (18)	−0.0069 (16)	−0.0029 (14)	−0.0095 (14)
C6	0.0532 (18)	0.0504 (16)	0.0454 (17)	0.0016 (14)	−0.0040 (14)	−0.0073 (13)
C7	0.0580 (19)	0.0580 (18)	0.0429 (17)	−0.0010 (15)	−0.0108 (14)	−0.0152 (14)
C8	0.0561 (18)	0.0514 (17)	0.0426 (17)	−0.0038 (14)	−0.0069 (14)	−0.0093 (13)
C9	0.076 (2)	0.077 (2)	0.050 (2)	0.0109 (19)	−0.0222 (17)	−0.0198 (17)
C10	0.094 (3)	0.080 (2)	0.052 (2)	0.020 (2)	−0.0315 (19)	−0.0110 (18)
C11	0.061 (2)	0.0577 (19)	0.060 (2)	0.0036 (16)	−0.0124 (16)	−0.0048 (16)
C12	0.073 (2)	0.064 (2)	0.054 (2)	0.0072 (18)	−0.0119 (17)	−0.0177 (16)
C13	0.067 (2)	0.065 (2)	0.0446 (18)	0.0085 (17)	−0.0135 (15)	−0.0092 (15)
C16	0.0449 (17)	0.0508 (17)	0.067 (2)	0.0010 (14)	−0.0148 (15)	−0.0147 (15)
C14	0.101 (4)	0.136 (5)	0.165 (5)	−0.045 (3)	−0.002 (4)	−0.044 (4)
C17	0.0483 (18)	0.0544 (18)	0.071 (2)	−0.0044 (15)	−0.0053 (15)	−0.0149 (16)
C15	0.150 (4)	0.088 (3)	0.106 (4)	0.035 (3)	−0.062 (3)	−0.009 (3)
C18	0.0488 (19)	0.0542 (19)	0.088 (3)	−0.0054 (15)	−0.0173 (17)	−0.0019 (17)
C19	0.0414 (17)	0.064 (2)	0.073 (2)	−0.0038 (15)	−0.0098 (15)	−0.0105 (17)
C20	0.055 (2)	0.0545 (19)	0.057 (2)	−0.0021 (16)	−0.0113 (15)	−0.0061 (15)
C21	0.0535 (19)	0.0526 (18)	0.0542 (19)	−0.0058 (15)	−0.0048 (14)	−0.0079 (14)
C22	0.063 (2)	0.0482 (17)	0.064 (2)	0.0012 (15)	−0.0132 (16)	−0.0077 (15)
C23	0.060 (2)	0.0426 (16)	0.0546 (18)	−0.0077 (15)	−0.0145 (15)	−0.0024 (13)
C24	0.066 (2)	0.0424 (16)	0.060 (2)	−0.0112 (15)	−0.0043 (16)	−0.0114 (14)
C25	0.058 (2)	0.0466 (17)	0.0555 (19)	−0.0084 (15)	0.0013 (15)	−0.0079 (14)
C26	0.056 (2)	0.0491 (17)	0.062 (2)	−0.0134 (15)	−0.0076 (16)	−0.0069 (15)
C27	0.074 (3)	0.056 (2)	0.101 (3)	−0.0117 (19)	−0.013 (2)	−0.030 (2)
C28	0.066 (2)	0.0502 (19)	0.094 (3)	0.0026 (16)	−0.0142 (19)	−0.0240 (17)
C29	0.065 (2)	0.068 (2)	0.106 (3)	−0.0165 (19)	0.002 (2)	−0.026 (2)
O6	0.0875 (18)	0.0769 (16)	0.0730 (16)	−0.0148 (14)	−0.0287 (13)	−0.0134 (13)
O7	0.0628 (16)	0.1024 (19)	0.0665 (16)	−0.0013 (14)	0.0093 (12)	−0.0209 (14)
O8	0.0543 (14)	0.0714 (15)	0.1018 (19)	−0.0079 (12)	−0.0103 (12)	−0.0396 (13)
O9	0.0468 (13)	0.0665 (14)	0.0915 (18)	−0.0104 (11)	0.0030 (12)	−0.0139 (12)
O10	0.0505 (14)	0.0747 (15)	0.0954 (18)	0.0078 (12)	−0.0081 (12)	−0.0283 (13)
N3	0.0462 (15)	0.0557 (15)	0.0569 (16)	−0.0011 (12)	−0.0034 (12)	−0.0148 (12)
N4	0.0431 (14)	0.0567 (15)	0.0786 (19)	−0.0027 (12)	−0.0045 (13)	−0.0298 (13)
C30	0.0544 (19)	0.0549 (18)	0.0478 (18)	0.0033 (14)	−0.0111 (14)	−0.0054 (14)
C31	0.109 (3)	0.0537 (19)	0.063 (2)	−0.0016 (19)	−0.042 (2)	−0.0020 (16)
C32	0.105 (3)	0.0507 (19)	0.066 (2)	−0.0030 (19)	−0.031 (2)	0.0012 (16)
C33	0.062 (2)	0.0560 (19)	0.061 (2)	0.0020 (16)	−0.0155 (16)	−0.0116 (16)
C34	0.064 (2)	0.081 (2)	0.061 (2)	−0.0079 (19)	−0.0262 (17)	0.0064 (18)
C35	0.054 (2)	0.066 (2)	0.064 (2)	−0.0082 (16)	−0.0190 (16)	0.0122 (16)
C36	0.0575 (19)	0.0482 (16)	0.0518 (19)	0.0014 (14)	−0.0139 (15)	−0.0003 (13)
C37	0.0550 (19)	0.0512 (17)	0.0451 (17)	0.0003 (14)	−0.0052 (14)	−0.0055 (13)
C38	0.066 (2)	0.063 (2)	0.057 (2)	0.0062 (17)	0.0098 (17)	0.0048 (16)
C39	0.068 (2)	0.064 (2)	0.058 (2)	0.0105 (17)	0.0030 (17)	−0.0051 (16)
C40	0.053 (2)	0.074 (2)	0.0459 (18)	−0.0055 (17)	0.0016 (15)	−0.0214 (16)
C41	0.058 (2)	0.078 (2)	0.0488 (19)	−0.0165 (18)	0.0018 (16)	−0.0015 (16)
C42	0.066 (2)	0.0537 (18)	0.0529 (19)	−0.0080 (16)	−0.0153 (16)	−0.0001 (14)
C43	0.176 (5)	0.074 (3)	0.129 (4)	−0.045 (3)	−0.059 (4)	0.000 (3)
C44	0.064 (2)	0.121 (3)	0.088 (3)	0.023 (2)	0.000 (2)	−0.032 (3)
C45	0.0386 (17)	0.0642 (19)	0.073 (2)	0.0001 (14)	−0.0091 (15)	−0.0206 (16)
C46	0.050 (2)	0.074 (2)	0.078 (2)	0.0040 (16)	−0.0140 (17)	−0.0315 (18)
C47	0.054 (2)	0.061 (2)	0.090 (3)	−0.0096 (16)	0.0123 (17)	−0.0329 (18)
C48	0.0487 (19)	0.0580 (19)	0.089 (3)	−0.0051 (15)	−0.0075 (17)	−0.0300 (17)
C49	0.0500 (18)	0.0514 (17)	0.0561 (19)	−0.0018 (15)	−0.0078 (14)	−0.0129 (14)
C50	0.0490 (18)	0.0498 (17)	0.0547 (18)	−0.0037 (14)	−0.0047 (14)	−0.0151 (14)
C51	0.0495 (18)	0.0523 (18)	0.0537 (18)	−0.0048 (14)	−0.0075 (14)	−0.0195 (14)
C52	0.0460 (17)	0.0503 (17)	0.0453 (16)	0.0008 (13)	−0.0061 (13)	−0.0160 (13)
C53	0.052 (2)	0.0508 (18)	0.086 (2)	−0.0072 (15)	−0.0064 (17)	−0.0266 (16)
C54	0.063 (2)	0.0503 (18)	0.092 (3)	0.0079 (17)	−0.0107 (19)	−0.0258 (17)
C55	0.0442 (18)	0.0594 (19)	0.0555 (19)	0.0022 (15)	−0.0043 (14)	−0.0200 (15)
C56	0.0440 (18)	0.0527 (17)	0.0474 (17)	−0.0068 (14)	0.0005 (13)	−0.0114 (13)
C57	0.0557 (19)	0.0435 (16)	0.0525 (18)	−0.0004 (14)	0.0014 (14)	−0.0117 (13)
C58	0.049 (2)	0.079 (2)	0.092 (3)	−0.0057 (19)	−0.0018 (18)	−0.020 (2)

### Geometric parameters (Å, º)

**Table:**

N1—C19	1.452 (4)	O6—C33	1.364 (4)
N1—C16	1.469 (3)	O6—C43	1.420 (5)
N1—C7	1.486 (4)	O7—C40	1.354 (4)
O1—C3	1.369 (4)	O7—C44	1.419 (4)
O1—C14	1.393 (6)	O8—C49	1.234 (3)
C1—C2	1.371 (5)	O9—C56	1.391 (3)
C1—C6	1.386 (4)	O9—C58	1.424 (4)
C1—H1A	0.9300	O10—C55	1.369 (3)
N2—C20	1.354 (4)	O10—C58	1.423 (4)
N2—C17	1.443 (4)	N3—C48	1.454 (4)
N2—C18	1.450 (4)	N3—C45	1.467 (4)
O2—C11	1.356 (4)	N3—C36	1.484 (4)
O2—C15	1.425 (4)	N4—C49	1.347 (4)
C2—C3	1.364 (5)	N4—C46	1.448 (4)
C2—H2A	0.9300	N4—C47	1.455 (4)
O3—C20	1.219 (4)	C30—C35	1.377 (4)
C3—C4	1.365 (5)	C30—C31	1.386 (4)
O4—C25	1.361 (4)	C30—C36	1.511 (4)
O4—C29	1.422 (4)	C31—C32	1.379 (4)
C4—C5	1.372 (4)	C31—H31A	0.9300
C4—H4A	0.9300	C32—C33	1.364 (4)
O5—C26	1.362 (4)	C32—H32A	0.9300
O5—C29	1.406 (4)	C33—C34	1.373 (4)
C5—C6	1.383 (4)	C34—C35	1.375 (4)
C5—H5A	0.9300	C34—H34A	0.9300
C6—C7	1.521 (4)	C35—H35A	0.9300
C7—C8	1.506 (4)	C36—C37	1.508 (4)
C7—H7A	0.9800	C36—H36A	0.9800
C8—C9	1.372 (4)	C37—C38	1.368 (4)
C8—C13	1.381 (4)	C37—C42	1.393 (4)
C9—C10	1.370 (4)	C38—C39	1.378 (4)
C9—H9A	0.9300	C38—H38A	0.9300
C10—C11	1.378 (5)	C39—C40	1.377 (4)
C10—H10A	0.9300	C39—H39A	0.9300
C11—C12	1.376 (4)	C40—C41	1.379 (4)
C12—C13	1.353 (4)	C41—C42	1.365 (4)
C12—H12A	0.9300	C41—H41A	0.9300
C13—H13A	0.9300	C42—H42A	0.9300
C16—C17	1.509 (4)	C43—H43A	0.9600
C16—H16A	0.9700	C43—H43B	0.9600
C16—H16B	0.9700	C43—H43C	0.9600
C14—H14A	0.9600	C44—H44A	0.9600
C14—H14B	0.9600	C44—H44B	0.9600
C14—H14C	0.9600	C44—H44C	0.9600
C17—H17A	0.9700	C45—C46	1.503 (4)
C17—H17B	0.9700	C45—H45A	0.9700
C15—H15A	0.9600	C45—H45B	0.9700
C15—H15B	0.9600	C46—H46A	0.9700
C15—H15C	0.9600	C46—H46B	0.9700
C18—C19	1.508 (4)	C47—C48	1.506 (5)
C18—H18A	0.9700	C47—H47A	0.9700
C18—H18B	0.9700	C47—H47B	0.9700
C19—H19A	0.9700	C48—H48A	0.9700
C19—H19B	0.9700	C48—H48B	0.9700
C20—C21	1.490 (4)	C49—C50	1.473 (4)
C21—C22	1.316 (4)	C50—C51	1.313 (4)
C21—H21A	0.9300	C50—H50A	0.9300
C22—C23	1.462 (4)	C51—C52	1.466 (4)
C22—H22A	0.9300	C51—H51A	0.9300
C23—C28	1.380 (4)	C52—C53	1.375 (4)
C23—C24	1.402 (4)	C52—C57	1.409 (4)
C24—C25	1.358 (4)	C53—C54	1.387 (4)
C24—H24A	0.9300	C53—H53A	0.9300
C25—C26	1.366 (4)	C54—C55	1.356 (4)
C26—C27	1.353 (5)	C54—H54A	0.9300
C27—C28	1.394 (5)	C55—C56	1.371 (4)
C27—H27A	0.9300	C56—C57	1.355 (4)
C28—H28A	0.9300	C57—H57A	0.9300
C29—H29A	0.9700	C58—H58C	0.9700
C29—H29B	0.9700	C58—H58A	0.9700
			
C19—N1—C16	108.0 (2)	C33—O6—C43	117.7 (3)
C19—N1—C7	109.9 (2)	C40—O7—C44	117.9 (3)
C16—N1—C7	111.7 (2)	C56—O9—C58	104.8 (2)
C3—O1—C14	118.8 (4)	C55—O10—C58	104.7 (2)
C2—C1—C6	121.7 (3)	C48—N3—C45	108.7 (2)
C2—C1—H1A	119.2	C48—N3—C36	110.7 (2)
C6—C1—H1A	119.2	C45—N3—C36	110.6 (2)
C20—N2—C17	127.6 (3)	C49—N4—C46	121.7 (2)
C20—N2—C18	120.0 (3)	C49—N4—C47	126.2 (3)
C17—N2—C18	112.3 (2)	C46—N4—C47	111.6 (2)
C11—O2—C15	118.7 (3)	C35—C30—C31	116.0 (3)
C3—C2—C1	121.0 (3)	C35—C30—C36	121.7 (3)
C3—C2—H2A	119.5	C31—C30—C36	122.3 (3)
C1—C2—H2A	119.5	C32—C31—C30	121.9 (3)
C2—C3—C4	118.5 (3)	C32—C31—H31A	119.0
C2—C3—O1	116.3 (3)	C30—C31—H31A	119.0
C4—C3—O1	125.3 (4)	C33—C32—C31	120.7 (3)
C25—O4—C29	106.4 (2)	C33—C32—H32A	119.6
C3—C4—C5	120.8 (3)	C31—C32—H32A	119.6
C3—C4—H4A	119.6	C32—C33—O6	125.4 (3)
C5—C4—H4A	119.6	C32—C33—C34	118.4 (3)
C26—O5—C29	106.1 (2)	O6—C33—C34	116.1 (3)
C4—C5—C6	121.9 (3)	C33—C34—C35	120.5 (3)
C4—C5—H5A	119.0	C33—C34—H34A	119.8
C6—C5—H5A	119.0	C35—C34—H34A	119.8
C5—C6—C1	116.2 (3)	C34—C35—C30	122.4 (3)
C5—C6—C7	122.2 (3)	C34—C35—H35A	118.8
C1—C6—C7	121.6 (3)	C30—C35—H35A	118.8
N1—C7—C8	112.9 (2)	N3—C36—C37	110.7 (2)
N1—C7—C6	110.2 (2)	N3—C36—C30	111.0 (2)
C8—C7—C6	110.7 (2)	C37—C36—C30	112.0 (3)
N1—C7—H7A	107.6	N3—C36—H36A	107.7
C8—C7—H7A	107.6	C37—C36—H36A	107.7
C6—C7—H7A	107.6	C30—C36—H36A	107.7
C9—C8—C13	115.7 (3)	C38—C37—C42	115.9 (3)
C9—C8—C7	121.1 (3)	C38—C37—C36	121.6 (3)
C13—C8—C7	123.2 (3)	C42—C37—C36	122.5 (3)
C10—C9—C8	123.4 (3)	C37—C38—C39	123.0 (3)
C10—C9—H9A	118.3	C37—C38—H38A	118.5
C8—C9—H9A	118.3	C39—C38—H38A	118.5
C9—C10—C11	119.3 (3)	C40—C39—C38	119.8 (3)
C9—C10—H10A	120.4	C40—C39—H39A	120.1
C11—C10—H10A	120.4	C38—C39—H39A	120.1
O2—C11—C12	116.8 (3)	O7—C40—C39	124.0 (3)
O2—C11—C10	125.0 (3)	O7—C40—C41	117.4 (3)
C12—C11—C10	118.3 (3)	C39—C40—C41	118.6 (3)
C13—C12—C11	121.1 (3)	C42—C41—C40	120.4 (3)
C13—C12—H12A	119.5	C42—C41—H41A	119.8
C11—C12—H12A	119.5	C40—C41—H41A	119.8
C12—C13—C8	122.3 (3)	C41—C42—C37	122.4 (3)
C12—C13—H13A	118.9	C41—C42—H42A	118.8
C8—C13—H13A	118.9	C37—C42—H42A	118.8
N1—C16—C17	110.8 (2)	O6—C43—H43A	109.5
N1—C16—H16A	109.5	O6—C43—H43B	109.5
C17—C16—H16A	109.5	H43A—C43—H43B	109.5
N1—C16—H16B	109.5	O6—C43—H43C	109.5
C17—C16—H16B	109.5	H43A—C43—H43C	109.5
H16A—C16—H16B	108.1	H43B—C43—H43C	109.5
O1—C14—H14A	109.5	O7—C44—H44A	109.5
O1—C14—H14B	109.5	O7—C44—H44B	109.5
H14A—C14—H14B	109.5	H44A—C44—H44B	109.5
O1—C14—H14C	109.5	O7—C44—H44C	109.5
H14A—C14—H14C	109.5	H44A—C44—H44C	109.5
H14B—C14—H14C	109.5	H44B—C44—H44C	109.5
N2—C17—C16	111.7 (3)	N3—C45—C46	111.1 (3)
N2—C17—H17A	109.3	N3—C45—H45A	109.4
C16—C17—H17A	109.3	C46—C45—H45A	109.4
N2—C17—H17B	109.3	N3—C45—H45B	109.4
C16—C17—H17B	109.3	C46—C45—H45B	109.4
H17A—C17—H17B	107.9	H45A—C45—H45B	108.0
O2—C15—H15A	109.5	N4—C46—C45	111.4 (3)
O2—C15—H15B	109.5	N4—C46—H46A	109.4
H15A—C15—H15B	109.5	C45—C46—H46A	109.4
O2—C15—H15C	109.5	N4—C46—H46B	109.4
H15A—C15—H15C	109.5	C45—C46—H46B	109.4
H15B—C15—H15C	109.5	H46A—C46—H46B	108.0
N2—C18—C19	110.7 (3)	N4—C47—C48	110.4 (3)
N2—C18—H18A	109.5	N4—C47—H47A	109.6
C19—C18—H18A	109.5	C48—C47—H47A	109.6
N2—C18—H18B	109.5	N4—C47—H47B	109.6
C19—C18—H18B	109.5	C48—C47—H47B	109.6
H18A—C18—H18B	108.1	H47A—C47—H47B	108.1
N1—C19—C18	111.7 (3)	N3—C48—C47	111.3 (3)
N1—C19—H19A	109.3	N3—C48—H48A	109.4
C18—C19—H19A	109.3	C47—C48—H48A	109.4
N1—C19—H19B	109.3	N3—C48—H48B	109.4
C18—C19—H19B	109.3	C47—C48—H48B	109.4
H19A—C19—H19B	107.9	H48A—C48—H48B	108.0
O3—C20—N2	121.0 (3)	O8—C49—N4	120.5 (3)
O3—C20—C21	121.3 (3)	O8—C49—C50	121.4 (3)
N2—C20—C21	117.7 (3)	N4—C49—C50	118.1 (3)
C22—C21—C20	121.2 (3)	C51—C50—C49	121.7 (3)
C22—C21—H21A	119.4	C51—C50—H50A	119.1
C20—C21—H21A	119.4	C49—C50—H50A	119.1
C21—C22—C23	127.7 (3)	C50—C51—C52	128.8 (3)
C21—C22—H22A	116.2	C50—C51—H51A	115.6
C23—C22—H22A	116.2	C52—C51—H51A	115.6
C28—C23—C24	118.7 (3)	C53—C52—C57	118.3 (3)
C28—C23—C22	118.4 (3)	C53—C52—C51	118.8 (3)
C24—C23—C22	122.9 (3)	C57—C52—C51	122.8 (3)
C25—C24—C23	118.1 (3)	C52—C53—C54	123.4 (3)
C25—C24—H24A	121.0	C52—C53—H53A	118.3
C23—C24—H24A	121.0	C54—C53—H53A	118.3
C24—C25—O4	128.6 (3)	C55—C54—C53	116.6 (3)
C24—C25—C26	122.2 (3)	C55—C54—H54A	121.7
O4—C25—C26	109.2 (3)	C53—C54—H54A	121.7
C27—C26—O5	128.0 (3)	C54—C55—O10	128.3 (3)
C27—C26—C25	121.6 (3)	C54—C55—C56	121.3 (3)
O5—C26—C25	110.4 (3)	O10—C55—C56	110.4 (3)
C26—C27—C28	117.0 (3)	C57—C56—C55	122.8 (3)
C26—C27—H27A	121.5	C57—C56—O9	128.9 (3)
C28—C27—H27A	121.5	C55—C56—O9	108.4 (3)
C23—C28—C27	122.4 (3)	C56—C57—C52	117.6 (3)
C23—C28—H28A	118.8	C56—C57—H57A	121.2
C27—C28—H28A	118.8	C52—C57—H57A	121.2
O5—C29—O4	108.0 (3)	O10—C58—O9	106.9 (3)
O5—C29—H29A	110.1	O10—C58—H58C	110.3
O4—C29—H29A	110.1	O9—C58—H58C	110.3
O5—C29—H29B	110.1	O10—C58—H58A	110.3
O4—C29—H29B	110.1	O9—C58—H58A	110.3
H29A—C29—H29B	108.4	H58C—C58—H58A	108.6
			
C6—C1—C2—C3	0.0 (6)	C35—C30—C31—C32	−0.3 (5)
C1—C2—C3—C4	−0.6 (6)	C36—C30—C31—C32	−179.0 (3)
C1—C2—C3—O1	178.4 (4)	C30—C31—C32—C33	0.9 (6)
C14—O1—C3—C2	171.9 (4)	C31—C32—C33—O6	−179.1 (3)
C14—O1—C3—C4	−9.2 (6)	C31—C32—C33—C34	−0.2 (6)
C2—C3—C4—C5	1.1 (5)	C43—O6—C33—C32	9.1 (6)
O1—C3—C4—C5	−177.8 (3)	C43—O6—C33—C34	−169.9 (4)
C3—C4—C5—C6	−0.9 (5)	C32—C33—C34—C35	−1.2 (6)
C4—C5—C6—C1	0.2 (5)	O6—C33—C34—C35	177.8 (3)
C4—C5—C6—C7	−177.6 (3)	C33—C34—C35—C30	1.9 (6)
C2—C1—C6—C5	0.2 (5)	C31—C30—C35—C34	−1.1 (5)
C2—C1—C6—C7	178.0 (3)	C36—C30—C35—C34	177.5 (3)
C19—N1—C7—C8	173.3 (2)	C48—N3—C36—C37	179.1 (2)
C16—N1—C7—C8	53.3 (3)	C45—N3—C36—C37	58.4 (3)
C19—N1—C7—C6	−62.3 (3)	C48—N3—C36—C30	−56.0 (3)
C16—N1—C7—C6	177.8 (2)	C45—N3—C36—C30	−176.6 (2)
C5—C6—C7—N1	−48.2 (4)	C35—C30—C36—N3	136.9 (3)
C1—C6—C7—N1	134.1 (3)	C31—C30—C36—N3	−44.5 (4)
C5—C6—C7—C8	77.5 (3)	C35—C30—C36—C37	−98.9 (3)
C1—C6—C7—C8	−100.2 (3)	C31—C30—C36—C37	79.7 (4)
N1—C7—C8—C9	−128.9 (3)	N3—C36—C37—C38	60.8 (4)
C6—C7—C8—C9	107.0 (3)	C30—C36—C37—C38	−63.5 (4)
N1—C7—C8—C13	53.1 (4)	N3—C36—C37—C42	−120.5 (3)
C6—C7—C8—C13	−71.1 (4)	C30—C36—C37—C42	115.1 (3)
C13—C8—C9—C10	1.9 (5)	C42—C37—C38—C39	0.4 (5)
C7—C8—C9—C10	−176.4 (3)	C36—C37—C38—C39	179.1 (3)
C8—C9—C10—C11	−1.2 (6)	C37—C38—C39—C40	−1.1 (5)
C15—O2—C11—C12	174.8 (3)	C44—O7—C40—C39	0.4 (5)
C15—O2—C11—C10	−6.0 (5)	C44—O7—C40—C41	179.9 (3)
C9—C10—C11—O2	−178.8 (3)	C38—C39—C40—O7	−179.7 (3)
C9—C10—C11—C12	0.5 (5)	C38—C39—C40—C41	0.8 (5)
O2—C11—C12—C13	178.7 (3)	O7—C40—C41—C42	−179.5 (3)
C10—C11—C12—C13	−0.7 (5)	C39—C40—C41—C42	0.1 (5)
C11—C12—C13—C8	1.4 (5)	C40—C41—C42—C37	−0.8 (5)
C9—C8—C13—C12	−1.9 (5)	C38—C37—C42—C41	0.5 (5)
C7—C8—C13—C12	176.2 (3)	C36—C37—C42—C41	−178.2 (3)
C19—N1—C16—C17	58.7 (3)	C48—N3—C45—C46	57.8 (3)
C7—N1—C16—C17	179.8 (2)	C36—N3—C45—C46	179.6 (2)
C20—N2—C17—C16	−127.5 (3)	C49—N4—C46—C45	−118.3 (3)
C18—N2—C17—C16	53.0 (3)	C47—N4—C46—C45	54.5 (4)
N1—C16—C17—N2	−56.2 (3)	N3—C45—C46—N4	−56.2 (3)
C20—N2—C18—C19	127.6 (3)	C49—N4—C47—C48	117.6 (3)
C17—N2—C18—C19	−52.7 (4)	C46—N4—C47—C48	−54.8 (4)
C16—N1—C19—C18	−59.7 (3)	C45—N3—C48—C47	−58.8 (3)
C7—N1—C19—C18	178.2 (2)	C36—N3—C48—C47	179.4 (2)
N2—C18—C19—N1	57.1 (4)	N4—C47—C48—N3	57.8 (3)
C17—N2—C20—O3	176.9 (3)	C46—N4—C49—O8	−2.7 (5)
C18—N2—C20—O3	−3.6 (5)	C47—N4—C49—O8	−174.3 (3)
C17—N2—C20—C21	−1.7 (5)	C46—N4—C49—C50	175.8 (3)
C18—N2—C20—C21	177.8 (3)	C47—N4—C49—C50	4.2 (5)
O3—C20—C21—C22	−17.8 (5)	O8—C49—C50—C51	23.0 (5)
N2—C20—C21—C22	160.8 (3)	N4—C49—C50—C51	−155.5 (3)
C20—C21—C22—C23	−175.9 (3)	C49—C50—C51—C52	170.9 (3)
C21—C22—C23—C28	170.3 (3)	C50—C51—C52—C53	−173.9 (3)
C21—C22—C23—C24	−7.0 (5)	C50—C51—C52—C57	1.8 (5)
C28—C23—C24—C25	−1.2 (4)	C57—C52—C53—C54	0.2 (5)
C22—C23—C24—C25	176.1 (3)	C51—C52—C53—C54	176.0 (3)
C23—C24—C25—O4	−178.3 (3)	C52—C53—C54—C55	−0.9 (5)
C23—C24—C25—C26	0.8 (5)	C53—C54—C55—O10	−179.8 (3)
C29—O4—C25—C24	177.8 (3)	C53—C54—C55—C56	1.5 (5)
C29—O4—C25—C26	−1.3 (4)	C58—O10—C55—C54	168.3 (3)
C29—O5—C26—C27	−178.9 (4)	C58—O10—C55—C56	−12.8 (3)
C29—O5—C26—C25	0.1 (4)	C54—C55—C56—C57	−1.5 (5)
C24—C25—C26—C27	0.6 (5)	O10—C55—C56—C57	179.6 (3)
O4—C25—C26—C27	179.9 (3)	C54—C55—C56—O9	178.3 (3)
C24—C25—C26—O5	−178.4 (3)	O10—C55—C56—O9	−0.6 (3)
O4—C25—C26—O5	0.8 (4)	C58—O9—C56—C57	−166.4 (3)
O5—C26—C27—C28	177.4 (3)	C58—O9—C56—C55	13.8 (3)
C25—C26—C27—C28	−1.5 (5)	C55—C56—C57—C52	0.8 (4)
C24—C23—C28—C27	0.4 (5)	O9—C56—C57—C52	−179.0 (3)
C22—C23—C28—C27	−177.0 (3)	C53—C52—C57—C56	−0.1 (4)
C26—C27—C28—C23	1.0 (5)	C51—C52—C57—C56	−175.8 (3)
C26—O5—C29—O4	−0.9 (4)	C55—O10—C58—O9	21.2 (3)
C25—O4—C29—O5	1.4 (4)	C56—O9—C58—O10	−21.6 (3)

### Hydrogen-bond geometry (Å, º)

**Table:**

*D*—H···*A*	*D*—H	H···*A*	*D*···*A*	*D*—H···*A*
C29—H29*A*···O10^i^	0.97	2.52	3.134 (5)	121
C44—H44*A*···O6^ii^	0.96	2.59	3.322 (5)	134
C47—H47*B*···O4^ii^	0.97	2.48	3.353 (5)	149
C53—H53*A*···O5^iii^	0.93	2.35	3.266 (4)	169

Symmetry codes: (i) −*x*, −*y*+1, −*z*+1; (ii) *x*+1, *y*, *z*; (iii) *x*+1, *y*+1, *z*.
